# Authors’ reply

**DOI:** 10.4103/0019-5545.64584

**Published:** 2010

**Authors:** Prabhat Sitholey, Vivek Agrawal, Amol Pargaonkar

**Affiliations:** Department of Psychiatry, CSM Medical University (Former K. G. Medical University), Lucknow - 226 003, India, E-mail: drvivekagarwal06@gmail.com; 1 Department of Psychiatry, Royal Perth Hospital, Perth, Australia

Sir,

We thank Dr. Helal and colleagues[1] for their interest in our work.[2] Our response to their comments are given below:

Autism is a well recognized psychiatric disorder. As in other psychiatric disorders, there is no blood test, neuro imaging or electroencephalogram (EEG) for the diagnosis of autism. Detailed psychiatric assessment and valid diagnostic criteria are used by trained professionals to make a diagnosis of autism. We did the same. In our case, observation was made by the psychiatrist, psychologist, nurses, parents, occupational therapist and autism trainer. The first author (PS) has worked in the United Kingdom with children and adolescents including those with autism. Although multidisciplinary assessment and management were the norms there, it did not imply that psychiatrists could not diagnose autism or that pediatricians were required essentially to make a diagnosis of autism. In India, diagnosis of autism is generally made by the psychiatrists but pediatricians or neurologists may also do so.

In our article, we have already expressed our views about improvement, remission and recovery.

Childhood autism rating score (CARS) was used only to rate the severity of autism.

It is true that about three quarter of the patients of autism also have co morbid mental retardation (MR). Trained professionals can distinguish between MR and autism. The autistic symptoms are specific and not shared by MR. Our case was diagnosed confidently as autistic on the basis of specific diagnostic criteria for autism and not because of developmental delays or low social/developmental quotients (SQ/DQ).

Although, SQ/DQ did improve, our case continued to be mentally retarded. This was a finding which was not used to judge recovery from autism.
